# Preparation of *Mycobacterium smegmatis* porin A (MspA) nanopores for single molecule sensing of nucleic acids

**DOI:** 10.52601/bpr.2021.210016

**Published:** 2021-10-31

**Authors:** Yuqin Wang, Pingping Fan, Shanyu Zhang, Shuanghong Yan, Shuo Huang

**Affiliations:** 1 State Key Laboratory of Analytical Chemistry for Life Sciences, School of Chemistry and Chemical Engineering, Nanjing University, Nanjing 210023, China; 2 Chemistry and Biomedicine Innovation Center (ChemBIC), Nanjing University, Nanjing 210023, China

**Keywords:** MspA nanopore, Prokaryotic expression, Affinity purification, Single molecule sensing

## Abstract

*Mycobacterium smegmatis* porin A (MspA), possessing a short (~0.6 nm) and narrow (~1.2 nm) constriction that enables its high spatial resolution of sensing, has emerged as an optimum choice for nanopore sequencing and nano-reactive sensing. However, prepared MspA nanopores cannot be obtained from any commercial vendors. We here provide a highly simplified protocol for MspA preparation. The protocol yields ~10 mg fully oligomerized protein per liter of culture and is straightforward to follow. With the prepared MspA nanopores, discrimination of immobilized ssDNA oligonucleotides can be performed, which serves as a demo application to demonstrate core procedures of single molecule sensing using MspA. This method is also in principle compatible with all other MspA mutants, which might merit the need to develop a variety of MspA based nano-reactive sensors.

## INTRODUCTION

*Mycobacterium smegmatis* porin A (MspA) is a conical shaped octameric biological nanopore. It contains a rigid 16-stranded β-barrel structure, which contributes to its thermal and chemical stability (Faller *et al.* 2004). It was reported that functional MspA pores stay active at a temperature up to 90 °C, within a pH range from 0–14, or in the presence of various denaturing agents (Heinz *et al.* 2003a). MspA also has an advantageous geometry for sensing. Briefly, the crystal structure reveals that the pore constriction measures just ~1.2 nm in diameter and 0.6 nm in length, fitting perfectly to the size of a single nucleotide on a strand of ssDNA (Derrington *et al.* 2010). This high spatial resolution has enabled MspA to achieve a single base resolution in the first demonstration of nanopore sequencing (Manrao *et al.* 2012). Moreover, in recent studies (Cao *et al.* 2019, 2021; Wang *et al.* 2020), highly engineered MspA nanopores were applied as a single molecule nanoreactors benefiting from its high spatial resolution of sensing and an overall conical geometry which fully amplifies perturbations caused by binding of an analyte, down to a single atomic ion. However, successful employment of engineered MspAs is only limited to few academic groups. This is in part because the reported methods of MspA preparation are not detailed enough to follow. By prokaryotic expression followed with purification by nickel affinity chromatography, our laboratory is capable of producing more than ten types of MspA mutants. A wide variety of corresponding applications including single molecule sensing (Cao *et al.* 2019, 2021; Wang *et al.* 2018, 2019b, 2020) and sequencing (Wang *et al.* 2019a; Yan *et al.* 2019; Zhang *et al.* 2020) were consequently reported. However, a detailed protocol describing how an MspA nanopore is prepared has not yet been disclosed, to the best of our knowledge.

### Development of MspA preparation methods

MspA belongs to a family of four very similar porins in *Mycobacterium smegmatis*. As the dominant porin in *Mycobacterium smegmatis* strains, MspA can be directly isolated from the bacterial lysates assisted with mild detergents at a room temperature. However, the yield and purity are not satisfying (Niederweis *et al.* 1999). By lysing *Mycobacterium smegmatis* with nonionic detergents at a temperature over 90 °C and purifying the treated lysate with anion exchange and size-exclusion chromatography (Heinz and Niederweis 2000), the yield is 20-fold increased, reporting a 20-μg protein production from a 1-L culture volume. For further structural studies by NMR or X-ray crystallography, optimized codons were introduced to the MspA gene as recombinant proteins when expressed by *E*. *coli* host cells. In this way, a yield of milligram MspA was achieved by multi-step chromatography, including anion exchange chromatography, hydrophobic interaction chromatography and size-exclusion chromatography (Heinz *et al.* 2003b; Sun *et al.* 2020). However, these preparation procedures are rather complicated, setting a high technical barrier for other interested researchers who may lack a required expertise of protein purifications.

Based on a previously published method developed by us (Cao *et al.* 2019, 2021; Wang *et al.* 2018, 2020), we present a highly simplified and effective protocol to prepare MspA and its functional mutants. This protocol is based on *E. coli* prokaryotic expression followed with one-step chromatography purification. A His-tag is incorporated into the recombinant protein to simplify the purification. During purification, the nonionic surfactant Genapol X-080 and a high lysing temperature are employed to promote the solvation of MspA. As an example of application, a nucleic acid immobilization assay was performed with the prepared MspA.

### Applications and advantages of the protocol

This tutorial protocol provides a step-by-step guide for MspA preparation and application. This preparation method can easily produce milligram quantities of MspA nanopores with a high stability, activity and reproducibility. Although not be demonstrated, other MspA mutants could in principle be prepared in the same way.

### Limitations of the protocol

The method for MspA preparation offers a high yield and purity but limited in the throughput. Because the employment of ÄKTA pure protein purification system only permits the purification of one type of MspA recombinant protein at a time. This may be inefficient to simultaneously screen a large variety of MspA mutants. A His-tag is required for nickel affinity purification. However, the addition of a His-tag to the C-terminus of the gene is not interfering with the sensing ability of MspA.

### Overview of the protocol

**Figure 1:**
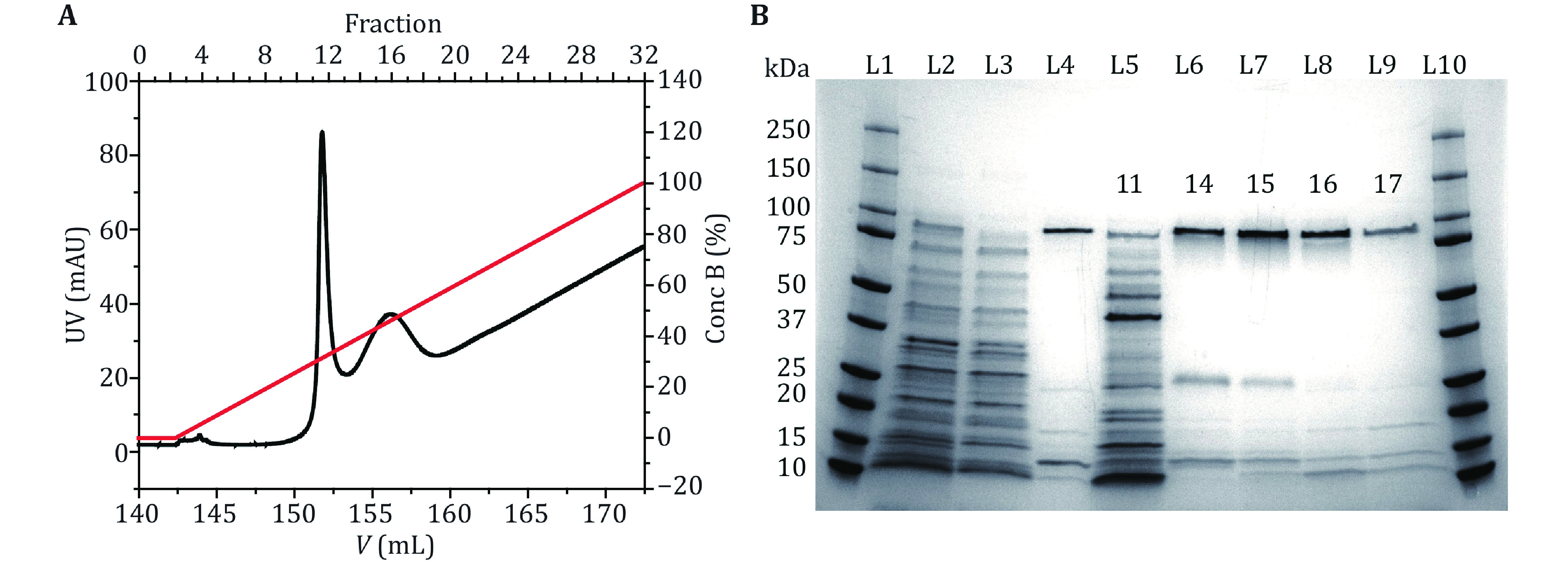
MspA purification and characterization. **A** The UV absorbance spectrum during the gradient elution of Ni-affinity chromatography. Two major peaks around the 11^th^ and the 16^th^ fractions were observed in the spectrum. Their identities were confirmed by gel electrophoresis. **B** MspA octamer characterized using SDS-polyacrylamide gel electrophoresis (4%–20% gradient gel). L1 and L10: precision plus protein standards (Bio-Rad). L2: the supernatant of the bacterial lysate. L3: the eluent of the bacterial lysate after column loading. L4: standard MspA sample. L5–L9: the eluted fractions in **A**. The index of the fraction was respectively marked on the gel. The gel results show that the first peak (fraction 11) corresponds to the eluent containing non-desired proteins. The second peak (fraction 14–17) corresponds to the eluent containing the desired MspA octamer. The impurity bands in lanes L6–L9 are some proteins of *E*. *coli* itself, which have non-specific adsorption with Ni-column

**Figure 2:**
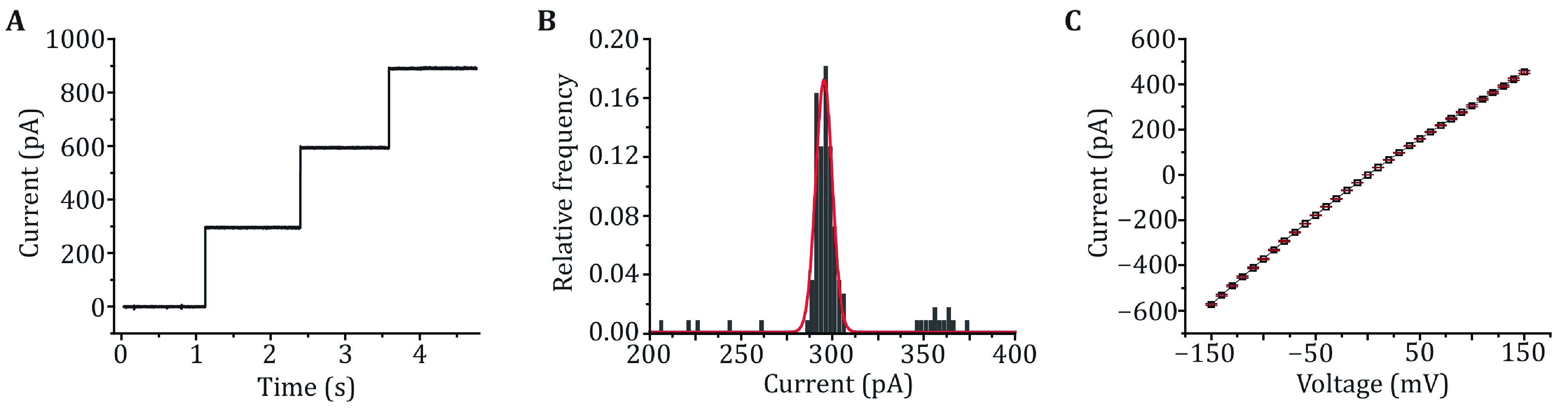
Single channel recording with MspA. **A** Successive channel insertions of MspA. A +100 mV voltage was continuously applied. Successive channel insertions would spontaneously happen, indicating that MspA obtained by this method show a high pore forming activity. **B** The histogram of open pore currents of MspA. A +100 mV voltage was applied during the measurement. The statistics were based on 50 nanopores (*N* = 100). **C** *I*–*V* curve of MspA. The statistics were based on 3 nanopores (*N* = 3). The mean values were shown with black squares and the error bars were shown with red lines. All measurements were performed in a 1.5 mol/L KCl buffer. MspA was added to *cis* to trigger the spontaneous pore insertion

**Figure 3:**
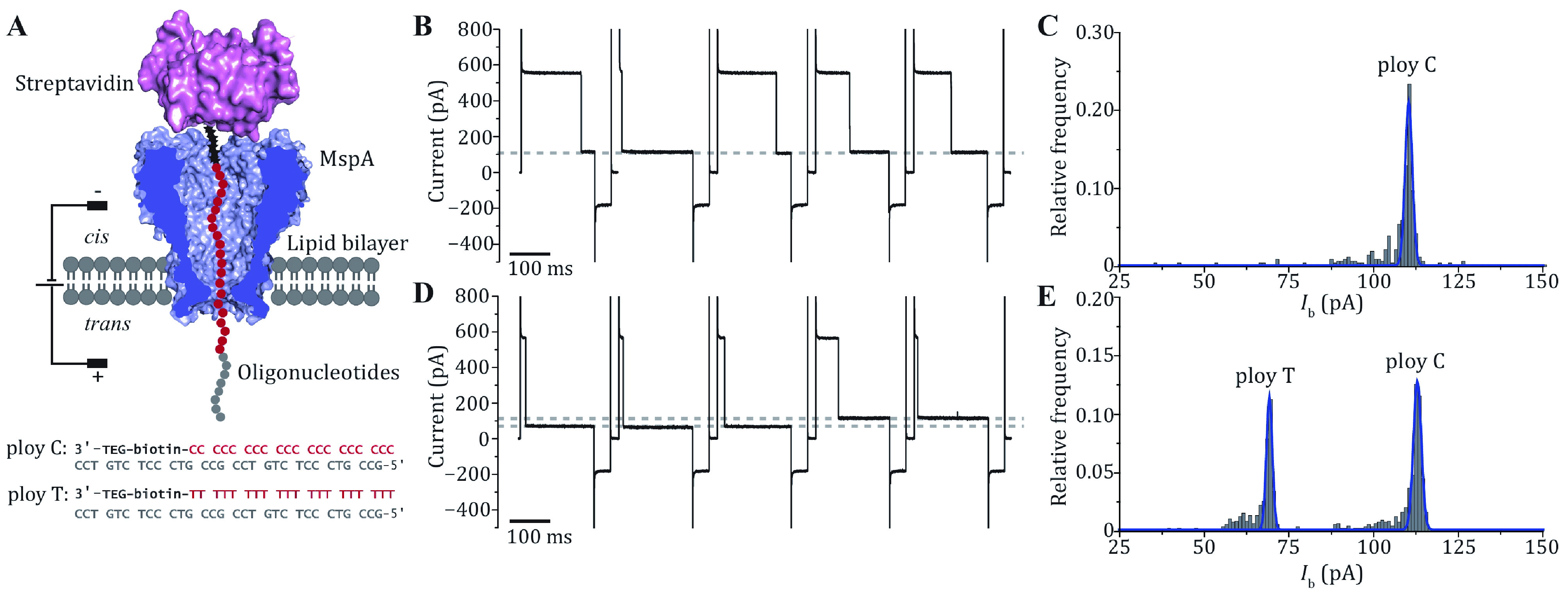
The DNA immobilization assay performed with MspA. **A** The schematic diagram of the measurement (top) and the sequence of the ssDNA oligonucleotides applied (bottom). Both oligonucleotides contain a 3’ biotin that can be tethered with a streptavidin stopper. During the measurement, the streptavidin-ssDNA complex can be electrophoretically captured by the pore and a residual current is reported. **B** Successive blockade events caused by the poly C-streptavidin complex. The blockade current level is indicated with the grey dotted line. **C** Histogram of the corresponding blockade current as demonstrated in **B**. Only a single peak is observed from the histogram. **D** Successive blockade events caused by the poly C-streptavidin complex and the poly T-streptavidin complex. The measurement was performed by subsequent addition of poly T-streptavidin complex to the measurement as described in **B**. Two blockade current levels were observed with highly distinguishable current levels. Both blockage levels are marked with grey dashed lines. **E** Histogram of corresponding blockade current as demonstrated in **D**. Two distinct peaks are observed respectively corresponding to nanopore reading of ploy C or ploy T. All measurements were performed in a 1.5 mol/L KCl buffer. MspA was added to *cis*. Oligonucleotides were added to *cis* with a final concentration of 200 nmol/L. A cyclic voltage protocol (+180 mV for 900 ms, –50 mV for 100 ms, 0 mV for 100 ms) was applied 1000 times to form a set of measurement. DNA blockade events were extracted using the “single channel research” option in Clampfit 10.7 (Molecular Devices)

In this protocol, the M2 MspA mutant (D90N/D91N/D93N/D118R/D134R/E139K), which has been widely applied in nanopore sequencing or as an engineering template to build nanoreactors, is employed as an example. For simplicity, this mutant is referred to as MspA throughout the paper, if not otherwise stated. The whole protocol consists of four steps, including MspA overexpression, purification, characterization by single channel recording and the DNA immobilization assay. Firstly, the gene coding for a monomeric MspA with a His-tag on its C-terminus is cloned into a pET-30a(+) plasmid. The plasmid is transformed to *E*. *coli* BL21 (DE3) for protein expression. After culture, the cells are lysed in the presence of Genapol X-080 at 60 °C and centrifuged to remove the cell debris. During cell lysis, the monomeric MspA spontaneously oligomerizes into a tightly bound octameric form. The produced MspA octamer can be purified by Ni-affinity column by elution with a concentration gradient of imidazole (Fig. 1A). The fractions containing the MspA octamer are collected and characterized by 4%–20% SDS PAGE (Fig. 1B). To evaluate the activity and repeatability of the prepared MspA nanopores, single molecule characterizations can be performed, including continuous pore insertion (Fig. 2A), open pore current statistics (Fig. 2B) and current–voltage (*I*–*V*) curve measurement (Fig. 2C). Finally, single molecule discrimination between ploy C and ploy T using MspA is demonstrated as a tutorial application (Fig. 3A–3E).

## EXPERIMENTAL DESIGN

### Construction of plasmids

The pET system is frequently applied for the expression of recombinant proteins in *E*. *coli* (Studier and Moffatt 1986). The pET vectors carry a chromosomal copy of the T7 RNA polymerase gene under lacUV5 control, which can be induced with isopropyl-β-D thiogalactoside (ITPG). The kanamycin resistance introduced to the pET vectors is useful to screen the transformed colonies. By enzymatic treatment with NdeI and HindIII, the gene coding for the MspA mutant containing a His-tag on its C-terminus is cloned to a pET-30a(+) plasmid. For simplicity, the successfully constructed plasmid is also shared on a public repository operated by Genescript (New Jersey). The readers can simply follow the link: https://www.molecularcloud.org/plasmid/M2-MspA/MC-0101191.html to request the plasmid.

### Prokaryotic expression

*E*. *coli* BL21 (DE3), a widely used T7 expression *E*. *coli* strain (Schlegel *et al.* 2012), is employed for protein expression. The plasmid coding for MspA is transformed into *E*. *coli* BL21 (DE3) and cultured in a lysogeny broth (LB) agar plate containing kanamycin. A single colony of transformed cells is then selected for culture in the LB broth. When the cell culture has reached an optical density at 600 nm (OD_600_) of 0.7, IPTG is added to induce protein expression.

### Cell lysis

The purpose of this step is to extract MspA from the cell membrane. Genapol X-080, a widely applied non-ionic detergent, is critical to lyse the cell membrane to assist MspA solubilization. Due to the thermal stability of MspA, a high temperature can as well be applied to remove a large proportion of impurity proteins which are not thermal stable.

### Nickel ion column affinity purification

His-Tag, composed of six histidines (Loughran and Walls 2011), is widely applied as a tag of recombinant proteins. It has a small molecular weight, which does not affect the structure and function of most target proteins. Commercial Nickel-affinity column immobilizes nickel ions on the chromatographic medium by chelating ligand nitazotriacetic acid (NTA) or iminodiacetic acid (IDA). Nickel ions can coordinate with the imidazole group of histidine, making the target protein with a His-Tag adsorbed on the affinity column. During purification, the binding buffer A, which contains 5 mmol/L imidazole, is firstly applied to elute nonspecifically adsorbed proteins from the column. Then a linear gradient of elution buffer B, which contains 500 mmol/L imidazole, is applied to elute MspA protein (Fig. 1A).

### The establishment of nanopore measurements

The measurement device consists of two custom poly-formaldehyde chambers separated by a ~20 μm-thick Teflon film. An aperture with a diameter of ~100 μm is located in the middle of the Teflon film. The aperture is firstly treated with 0.5% (*v*/*v*) pentane solution of hexadecane and air dried to fully evaporate the pentane. During the measurements, both chambers are filled with KCl electrolyte buffer. A pair of Ag/AgCl electrodes is applied to generate the desired voltages across the membrane. After the addition of a drop of lipid to each chamber, the buffer level in one of the chambers was lowered and restored repetitively to form the lipid bilayer. As a transmembrane protein, MspA in the buffer would spontaneously insert into the lipid bilayer, forming the only channels connecting the two chambers. To avoid further pore insertions, the buffer should be quickly exchanged with fresh ones. All electrophysiology measurements are then performed with an Axopatch 200B patch clamp amplifier paired with a Digidata 1550B digitizer. Recordings are sampled at 25 kHz and low-pass filtered with a 1-kHz cutoff frequency.

### Discrimination of immobilized oligonucleotide with MspA

As a demo of application, we performed single molecule discrimination of two terminal biotin modified ssDNA oligonucleotides, namely ploy C (3’-TEG-biotin-C_22_ CCT GTC TCC CTG CCG CCT GTC TCC CTG CCG-5’) and ploy T (3’-TEG-biotin-T_22_ CCT GTC TCC CTG CCG CCT GTC TCC CTG CCG-5’). The biotin-tags allow the oligonucleotides to be tethered with a streptavidin stopper, forming a streptavidin-DNA complex. Assisted with the streptavidin, which is too large to translocate through the pore, the oligonucleotides can be electrophoretically immobilized in the pore, reporting a long residing residual current (Fig. 3A) (Manrao *et al.* 2011; Stoddart *et al.* 2009). Consequently, poly C and ploy T, which produces different residual currents, are distinguished (Fig. 3E).

## PROCEDURE

### Prokaryotic expression of MspA [TIMING ~40 h]

*Step 1*: *Plasmid transformation*

Thaw 100 μL *E. coli* BL21 (DE3) competent cells on ice for 30 min. Mix 1 μL 100 ng/μL MspA plasmid into 100 μL competent cells. Gently mix by finger flicking the bottom of the tube for several times. Incubate the mixture on ice for 30 min. Heat shock the cells on a metal bath set at 42 °C for 90 s and quickly place the cells back on ice for 3–5 min.

**[CRITICAL STEP]** Competent cells are fragile, so all operations should be done gently and avoid violent shaking.

**[CRITICAL STEP]** In order to avoid introduction of other molds or fungus, operations shall be performed on a biosafety cabinet and all equipment should be sterilized by UV exposure prior to each use.

*Step 2*: *Spreading plate*

Place all transformed cells on a LB agar plate containing kanamycin. Spread evenly with a glass drigalski spatula (triangle-shaped). Invert the petri dish and culture in a thermostatic incubator at 37 °C for 18–20 h.

**[CRITICAL STEP]** Operations shall be performed in a biosafety cabinet and all equipment should be sterilized by UV exposure prior to each use.

*Step 3*: *Culturing a single colony*

Pick a single colony from the LB agar plate with a 200 μL-tip and add it to a flask containing 100 mL LB broth. Top the flask with a breathable sealing film to maintain the oxygen level in the culture without getting environmental contaminations. Culture in an oscillating incubator at 200 r/min for 5–6 h at 37 °C.

**[CRITICAL STEP]** A desired spread plate should contain a countable number of isolated colonies evenly distributed on the plate. Carefully pick a colony and avoid introducing surrounding colonies.

**[CRITICAL STEP]** Operations shall be performed on a biosafety cabinet and all equipment should also be sterilized by UV before use.

**[? TROUBLESHOOTING]**

*Step 4*: *Induction expression of target fusion protein*

Until the cells are grown to an OD_600_ = 0.7, cool the culture down to the room temperature. Spare 1 mL cell culture for sequencing characterization. Add 100 μL 1 mol/L IPTG solution to the remaining cell culture to activate protein expression. Culture at 200 r/min for 12 h at 16 °C.

**[CRITICAL STEP]** If the bacteria grow too dense, the cells will inhibit the protein expression.

**[CRITICAL STEP]** Ensure that the plasmid sequencing result is correct before proceeding with subsequent purifications.

**[CRITICAL STEP]** Operations shall be performed on a biosafety cabinet and all equipment should be sterilized by UV prior to each use.

**[? TROUBLESHOOTING]**

### Purification and characterization of MspA [TIMING ~7 h]

*Step 5*: *Cell lysis*

Centrifuge the cells at 4000 r/min, for 20 min at 4 °C to remove the LB broth. Resuspend the cell pellet in a 10 mL lysis buffer and stir well. Heat at 60 °C in a metal bath for 20 min and keep it on ice for 10 min. Centrifuge the cells at 13,000 r/min for 40 min at 4 °C to remove nuclei, cell debris, and unbroken cells. Filter the protein supernatant with a 0.2-μm syringe filter to avoid clogging the Ni-affinity column.

*Step 6*: *Purification of MspA with Ni-affinity chromatography*

Set the flow rate at 1 mL/min. Elute the nickel column with 5 column volumes of Milli-Q, 5 column volumes of elution buffer B and 10 column volumes of binding buffer A successively. Load the protein supernatant manually. Set the flow rate at 4 mL/min. Remove the unbound protein with binding buffer A until the UV value is stable. Set the flow rate at 1 mL/min and collect the eluent at a rate of 1 mL/tube. Elute MspA protein using a linear gradient of 0–100% elution buffer over 30 column volumes within 30 min. Two major peaks will be observed in the spectrum (Fig. 1A).

**[CRITICAL STEP]** Solutions should be processed with vacuum filtration to avoid clogging the column.

**[? TROUBLESHOOTING]**

*Step 7*: *Characterization of MspA with polyacrylamide gel electrophoresis*

Collect the samples including the supernatants of the bacterial lysate before and after loading to Ni-affinity chromatography, the 11^th^, 14^th^, 15^th^, 16^th^ and 17^th^ tubes of protein eluent. Load the samples on a 4%–20% SDS-polyacrylamide gel and then run for 27 min with a +200-V applied potential (Fig. 1B). Stain the gel with Commassie blue fast staining solution for 15–20 min and visualize it with gel imager system. Theoretically, the band around 100 kDa corresponds to the MspA octamer. After gel electrophoresis, measure MspA concentration using nanodrop and store it at −80 °C for future uses.

**[? TROUBLESHOOTING]**

### Single channel recording of MspA [TIMING ~15 h]

*Step 8*: *Preparation of Ag/AgCl electrodes*

Solder a male pin and a piece of silver wire (~2 cm in length) at the two ends of a wire separately. Seal the welding joints with shrinkable tubes. Soak the silver wire in 30% (*v*/*v*) NaOCl (diluted in water) overnight until the silver surface is greyish black. Rinse the electrodes with Milli-Q and ethanol, air-dry with nitrogen gas and store away from light.

*Step 9*: *Assemble of homemade measurement device*

The measurement device consists of two mirror symmetrical modules, separated by a Teflon film. Each module is provided with a sample chamber (~500 μL) and three electrode holes. Breakdown an aperture (~100 μm in diameter) in the Teflon film using a homemade arc generator. Sandwich the Teflon film between the two modules with glues and fasten the two modules together with screws. Rest the assembly overnight until the glue has fully set. To enrich phospholipid molecules, point-coat a small amount of hexadecane solution around the hole with a capillary under the microscope before use.

**[CRITICAL STEP]** The both sides of the aperture should be tapped with hexadecane solution.

*Step 10*: *Construction of detection system*

Use AxoPatch 200B Amplifier and Axon Digidata 1500B for measurements. To shield mechanical and electromagnetic noise, place the measurement device inside a homemade Faraday cage, mounted on an optical table. Connect male pin ends of a pair of Ag/AgCl electrodes to Axopatch 200B Amplifier probes. Insert silver wire ends of the two Ag/AgCl electrodes to one of electrode holes of the two chambers. Conventionally, the chamber electrically grounded is defined as *cis*, and the opposing chamber is defined as *trans*.

*Step 11*: *Formation of lipid bilayer*

Add 500 μL KCl buffer to the *cis* and *trans* chambers separately. Respectively add a drop of lipid to both chambers using capillary. Switch to a +20 mV-constant voltage. Raise and drop the buffer in the chamber back and forth using a pipette. Repeat several times until the current changes from 19 nA (a short circuit) to 0 pA (a broken circuit). Switch to a triangle wave voltage protocol (Peak amplitude: ±50 mV, frequency: 5 Hz) to check the quality of the lipid bilayer. The bilayer capacitance can be determined according to $ C=I/(dV/dt) $, where “*I*” stands for the absolute current magnitude of the square wave signal. An ideal lipid bilayer usually generates a standard square wave current with amplitude range from 50 to 100 pA.

**[? TROUBLESHOOTING]**

*Step 12*: *Single channel characterization of MspA*

Add MspA nanopore to *cis* and stir well. Switch to a +100 mV-constant voltage. Record the process of successive pore insertions (Fig. 2A). When the current is unstable, break the lipid bilayer and pull it up again. Repeat until a total of about 100 pores have been recorded to form a statistic (Fig. 2B).

**[? TROUBLESHOOTING]**

*Step 13*: *Insertion of single MspA nanopore in the lipid bilayer*

Switch to a +20 mV-constant voltage. Add MspA nanopore to *cis* and stir well. Switch to a square wave voltage protocol (Peak amplitude: ±200 mV, frequency: 1 Hz) to facilitate pore insertion. Wait until a current stepping occurs. Switch to a 0 mV-constant voltage and adjust the current to zero. Switch to a +20 mV-constant voltage. If the open pore current is stable and appropriate (~64 pA), quickly exchange the buffer in *cis* with fresh KCl buffer for about 10 times to avoid further pore insertions. Switch to a triangle voltage (increasing from −150 mV to 150 mV at intervals of 10 mV and each voltage lasts for 500 ms). Record the *I*–*V* curve of MspA (Fig. 2C).

**[? TROUBLESHOOTING]**

### Single molecule sensing of immobilized DNA oligonucleotide [TIMING ~1 h]

*Step 14*: *Recording background signal*

Switch to a static blockade voltage protocol (+180 mV for 900 ms, −50 mV for 100 ms, 0 mV for 100 ms, circular 500 times). Record the current.

**[? TROUBLESHOOTING]**

*Step 15*: *Sensing of immobilized poly C*

Premix 3.8 μL poly C-biotin (100 μmol/L) and 20 μL streptavidin (1 mg/mL, 18.9 μmol/L). Switch to a +20 mV-constant voltage. Add 6.25 μL mixture of poly C-biotin and streptavidin in *cis* and stir well. Switch to the static blockade voltage protocol (+180 mV for 900 ms, −50 mV for 100 ms, 0 mV for 100 ms, circular 1000 times) and record the current (Fig. 3B).

**[? TROUBLESHOOTING]**

*Step 16*: *Simultaneously sensing of immobilized poly C and ploy T*

Premix 3.8 μL poly T-biotin (100 μmol/L) and 20 μL streptavidin (1 mg/mL, 18.9 μmol/L). Switch to a +20 mV-constant voltage. Add 6.25 μL mixture of poly T-biotin and streptavidin in *cis* and stir well. Switch to the static blockade voltage protocol (+180 mV for 900 ms, −50 mV for 100 ms, 0 mV for 100 ms, circular 1000 times) and record the current (Fig. 3D).

**[? TROUBLESHOOTING]**

Troubleshooting advice can be found in Table 1.

**Table 1:** Troubleshooting table

Steps	Problem	Possible reason	Solution
3	No colonies are gown	The competent cells are inactive	Use alive competent cells
4	The sequencing result is wrong	Experimental apparatus has contaminates of molds or fungus	Increase the time of UV sterilization and standardize experimental operations
6, 7	The yield is low	The affinity column has lost its affinity to the desired protein	Replace a new affinity column
11	The capacitance of the bilayer is too high	Hexadecane is too much	Redo the experiment and add less hexadecane in Step 8
11	The capacitance of the bilayer is too low	Hexadecane is insufficient	Redo the experiment and add more hexadecane in Step 8
11	The current response to a triangle wave voltage protocol deviates from the standard square wave	Hexadecane is too much or there is a leakage current	Redo the experiment and add less hexadecane in Step 8
12, 13	No MspA is inserted	MspA is insufficient	Add more MspA to *cis*
13–16	More than one pore is inserted	The buffer is exchanged incompletely	Re-initiate the experiment and increase the times of buffer exchange
13–16	The lipid bilayer is unstable	The measurement time is too long	Add lipid in the two chambers and switch to a low voltage for a few minutes or restart single channel recording

[**TIMING**]

Step 1: Plasmid transformation (~1 h)

Step 2: Cell culture in LB agar plate (18–20 h)

Step 3: Selection of single colony (~1 h); Cell culture in LB broth (5–6 h)

Step 4: Induction expression (~12 h)

Step 5: Cell lysis (~2 h)

Step 6: MspA purification (~3 h)

Step 7: Gel electrophoresis (~2 h)

Step 8: Preparation of Ag/AgCl electrodes (~1 h); Immersion in 30% (*v*/*v*) NaOCl (12 h)

Step 9: Preparation of measurement device (~1 h); Gel solidification (12 h)

Step 10: Construction of detection system (30 min)

Step 11: Formation of lipid bilayer (30 min)

Step 12: Single channel characterization of MspA (~1 h)

Step 13: Insertion of single MspA nanopore (~1 h)

Step 14: Recording background signal (10 min)

Step 15: Sample preparation (5 min); Recording signals of ploy C sensing (20 min)

Step 16: Sample preparation (5 min); Recording signals of ploy C and ploy T sensing (20 min)

## ANTICIPATED RESULTS

Generally, the prepared MspA spontaneously oligomerize into an octameric form. Monomeric MspA is however rarely seen. During a gradient elution (Step 6), non-specific proteins and MspA octamers will be eluted in a sequential order. Two peaks, respectively around the 11^th^ and the 16^th^ fraction are expected to appear in the UV absorbance spectrum (Fig. 1A). Their identities are subsequently confirmed based on the gel electrophoresis results in Fig. 1B (Step 7). In denaturing polyacrylamide gels, the octameric MspA migrates equivalently to that of a molecular mass of 100 kDa, which could be found between the 14^th^ and the 17^th^ fractions.

The quality of the prepared MspA can be checked by single channel recording. After adding MspA to *cis*, spontaneous and successive pore insertion could be observed (Step 12, Fig. 2A). This indicates that MspA expressed in *E*. *coli* still has high pore-forming activity. The open pore current of each MspA shows a high consistency, the distribution of which follows a Gaussian fitting with a mean value of 295.39 pA at +100 mV (Step 12, Fig. 2B). The *I*–*V* curve acquired from a single MspA shows a general linear correlation between current and voltage (Step 13, Fig. 2C).

In the example of application, when attached to streptavidin and held within MspA, poly C and poly T can be clearly distinguished by reporting different residual currents ($ {I}_{\text{b}} $) (Fig. 3A). The open pore current ($ {I}_{\text{o}} $) of single MspA nanopore is usually around 554.20 pA at +180 mV. When ploy C-streptavidin complex was added to *cis*, successive blockade events with the same residual current was reported (Step 15, Fig. 3B). The distribution of $ {I}_{\text{b}} $ follows a Gaussian distribution centered at 110.35 pA (Fig. 3C). When ploy T-streptavidin complex was further added to *cis*, deeper blockades were observed (Step 16, Fig. 3D). Histogram plots of $ \Delta I $ also show two peaks centered at 69.19 pA and 112.90 pA, respectively corresponding to reading of ploy T and ploy C (Fig. 3E).

## MATERIALS

### Reagents

• MspA plasmid (Genscript, customized product)

• *E. coli* BL21 (DE3) competent cell (Biomed, cat. no. CC0703)

• Kanamycin (Beyotime, cat. no. ST008)

• IPTG (Solarbio, cat. no. I8070)

• LB broth (Hopebio, cat. no. 140665)

• LB Agar (Hopebio, cat. no. 140664)

• NaH_2_PO_4_ (Aladdin, cat. no. S817796)

• EDTA (Sigma-Aldrich, cat. no. 324504)

• NaCl (Aladdin, cat. no. C111535)

• KCl (Aladdin, cat. no. P112133)

• NaOCl (Guangdong Guanghua Sci-Tech Co., Ltd., cat. no.1.01412.018)

**[CAUTION!]** NaOCl is corrosive and hazardous to environment. Handle with protective gloves.

• HEPES (Shanghai Yuanye Bio-Technology, cat. no. S16013)

• Genapol X-80 (Sigma, cat. no. G6923)

• Imidazole (Solarbio, cat. no. I8090)

**[CAUTION!]** Imidazole is acutely toxic. Handle with protective gloves.

• Ethanol (Ghtech, cat. no. 1.17113.033)

**[CAUTION!]** Ethanol is flammable. Avoid flames and handle in a fume hood.

• Hexadecane (Sigma-Aldrich, cat. no. V900147)

• Silicone oil AR20 (Sigma-Aldrich, cat. no. 10838)

• Pentane (Sigma-Aldrich, cat. no. 236705)

**[CAUTION!]** Hexane is acutely toxic and flammable. Avoid flames and handle in a fume hood.

• 1,2-diphytanoyl-sn-glycero-3-phosphocholine (Avanti Polar Lipids, cat. no. 850356)

• Poly T-TEG-biotn (Sangon Biotech, customized product)

• Poly C-TEG-biotn (Sangon Biotech, customized product)

• Streptavidin (New England Biolabs, cat. no. N7021S)

• Ultrapure water (Milli-Q)

• 4%−20% Mini-PROTEAN TGX Gel (Bio-Rad, cat. no. 4561083S)

• Precision Plus Protein™ Dual Xtra Standards (Bio-Rad, 1610374)

• SDS-PAGE sample loading buffer (Beyitime, cat. no. P0015F)

• Coomassie blue fast staining solution (Beyitime, cat. no. P0017)

### Reagent setup

• Kanamycin solution (10 mg/mL). Dissolve 0.05 g Kanamycin in 5 mL Milli-Q and stir well. Store at −20 °C.

• IPTG solution **(**1 mol/L). Dissolve 0.4766 g IPTG in 2 mL Milli-Q and stir well. Store at −20 °C.

• LB broth. Dissolve 2.5 g LB broth powder in 100 mL Milli-Q. Autoclave at 121 °C for 15 min and then cool at room temperature. Transfer it to biosafety cabinet. Add 100 μL 10 mg/mL kanamycin solution and stir well. This solution can be stored at 4 °C for one week.

• LB agar plate. Dissolve 3.8 g LB agar in 100 mL Milli-Q. Autoclave at 121 °C for 15 min and then cool to 55 °C at room temperature. Transfer it to biosafety cabinet. Add 100 μL 10 mg/mL kanamycin solution and stir well. Pour the agar evenly into the petri dishes (~25 mL for each plate) and allow each plate to cool until the agar sets and hardens (~10 min). Cover the lid and seal it with the parafilm. This medium can be stored at 4 °C for two weeks.

• Lysis buffer (100 mmol/L Na_2_HPO_4_/NaH_2_PO_4_, 0.1 mmol/L EDTA, 150 mmol/L NaCl, 0.5% (*w*/*v*) Genapol X-100, pH 6.5). Dissolve 6.0 g NaH_2_PO_4_, 14.61 mg EDTA, 4.38 g NaCl in 450 mL Milli-Q and stir well. Adjust the pH to 6.5 with HCl. Bring the volume to 500 mL with Milli-Q. Add 2.5 mL Genapol X-80 and stir well. Store at room temperature.

• Binding buffer A (0.5 mol/L NaCl, 20 mmol/L HEPES, 5 mmol/L imidazole, 0.5% (*w*/*v*) Genapol X-080, pH = 8.0). Dissolve 14.61 g NaCl, 2.383 g HEPES, 0.17 g imidazole, 2.5 mL Genapol X-080 in 400 mL Milli-Q and stir well. Adjust the pH to 8.0 with NaOH. Bring the volume to 500 mL with Milli-Q. Filter the buffer in the vacuum with a membrane filter. Store at room temperature.

• Elution buffer B (0.5 mol/L NaCl, 20 mmol/L HEPES, 500 mmol/L imidazole, 0.5% (*w*/*v*) Genapol X-080, pH = 8.0). Dissolve 14.61 g NaCl, 2.383 g HEPES, 17.0 g imidazole, 2.5 mL Genapol X-080 in 400 mL Milli-Q and stir well. Adjust the pH to 8.0 with NaOH. Bring the volume to 500 mL with Milli-Q. Filter the buffer in the vacuum with a membrane filter. Store at room temperature.

• Hexadecane (0.5% (*v*/*v*)). Add 5 μL hexadecane in 995 μL pentane. Stir well and seal the sealing film. Store at −20 °C.

• Lipid (5 mg/mL). Dissolve 25 mg 1,2-diphytanoyl-sn-glycero-3-phosphocholine in 5 mL pentane. Stir well and seal the sealing film. Store at −20 °C.

• KCl buffer (1.5 mol/L KCl, 10 mmol/L HEPES, pH = 7.0). Dissolve 55.9125 g KCl and 1.1915 g HEPES in 400 mL Milli-Q. Adjust the pH to 7.0 with NaOH. Bring the volume to 500 mL with Milli-Q. Filter the buffer in the vacuum with a membrane filter. Store at room temperature.

### Consumables

• Sliver wire (Alfa Aesar, cat. no. 42487)

• AMPLIMITE HDP-20 Male pine (TE Connectivity, cat. no. 66506-4)

• Teflon film (Thickness: 20 µm)

• Syringe filter (Pall corporation, cat. no. 4612)

• Membrane filter (Whatman, cat. no. 7001 004)

• Arc generator (homemade)

• Measurement device (homemade)

• Faraday cage (homemade)

### Equipments

• Ultrapure Water Systems (Millipore)

• Autoclave (Zealway, cat. no. GI54DWS)

• Biosafety cabinet (Airtech, cat. no. BSC-1000IIA2)

• Digital heating cooling drybath (Thermofisher, cat. no. 88880030)

• Thermostatic incubator (Senxin, cat. no. DRP-9052)

• Full temperature double layer oscillating incubator (PeiYing, cat. no. HZQ-F100)

• Sorvall ST 8R small benchtop bentrifuge (Thermofisher, cat. no. 75007204)

• ÄKTA pure protein purification system (Cytiva)

• HisTrap^TM^HP nickel ion affinity column (GE Healthcare)

• Mini vertical electrophoresis apparatus (Baygene, cat. no. BG-verMINI)

• BG-verMINI electrophoresis dystem (Baygene, cat. no. BG-Power600h)

• G:BOX F3 gel documentation dystem (Syngene)

• Stereo microscope (Nanjing Jiangnan Novel Optics Co., Ltd, cat. no. JSZ6S)

• Axopatch 200B amplifier (Molecular Devices)

• Axon digidata 1550B (Molecular Devices)

• Optical table (Jiangxi Liansheng technology Co., Ltd., cat. no. ZDT09-06)

## Abbreviations

*E. coli*　　*Escherichia coli*

IPTG　　 Isopropyl-β-D thiogalactoside

LB　　　 Lysogeny broth

MspA　　*Mycobacterium smegmatis* porin A

## Conflict of interest

Yuqin Wang, Pingping Fan, Shanyu Zhang, Shuanghong Yan and Shuo Huang declare that they have no conflict of interest.
